# 3D mapping of nanoscale crosslink heterogeneities in microgels†
†Electronic supplementary information (ESI) available: Detailed synthesis and molecular characterization of compounds; hydrogels and microgels preparation details; additional DLS and SEM results; video of dye diffusion from hydrogel matrix. See DOI: 10.1039/c8mh00644j


**DOI:** 10.1039/c8mh00644j

**Published:** 2018-09-05

**Authors:** Apostolos A. Karanastasis, Yongdeng Zhang, Gopal S. Kenath, Mark D. Lessard, Joerg Bewersdorf, Chaitanya K. Ullal

**Affiliations:** a Department of Materials Science and Engineering , Rensselaer Polytechnic Institute , Troy , New York 12180 , USA . Email: ullalc@rpi.edu; b Department of Cell Biology , Yale University , New Haven , CT 06520 , USA; c Department of Biomedical Engineering , Yale University , New Haven , CT 06520 , USA

## Abstract

The majority of swollen polymer networks exhibit spatial variations in crosslink density. These spatial heterogeneities are particularly important in colloidal gel particles, or microgels, where they manifest themselves on the nanoscale and impact mechanical and transport properties. Despite their importance, the real space nanostructure of these heterogeneities at the individual particle level has remained elusive. Using state of the art super-resolution microscopy known as Whole cell 4Pi Single Molecule Switching Nanoscopy (W-4PiSMSN) we demonstrate 3D nanoscale mapping of spatial crosslink heterogeneities in a model system of poly(*N*-isopropylacrylamide) colloidal gel particles containing a novel fluorophore tagged crosslinker. We reveal the presence of higher crosslink density clusters embedded in a lower crosslink density matrix within the core of individual microgel particles, a phenomenon that has been predicted, but never been observed before in real space. The morphology of the clusters provides insight into the kinetics of microgel formation. This study also provides proof-of-concept 3D super-resolution imaging of spatial heterogeneities in bulk hydrogels.

## Abstract 2

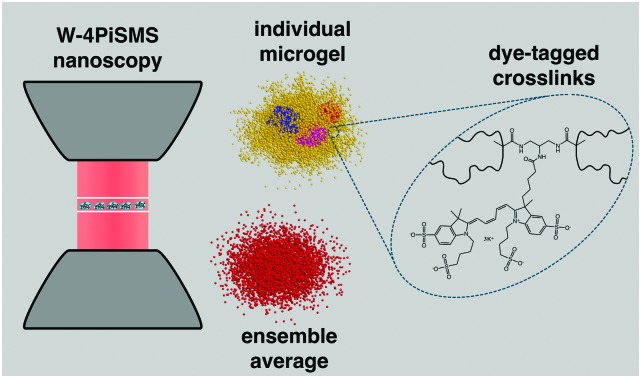
The majority of gels exhibit nanoscale spatial variations in crosslink density. We present the first 3D super-resolution microscopy images of dye tagged cross-link distributions in microgels and hydrogels. The morphology of nanoscale features never imaged previously in microgels, are revealed.

##

Conceptual insights

A long-standing challenge in the areas of networked polymers and stimuli responsive gels is that the spatial distribution of crosslinks in most hydrogels and colloidal gels particles is not uniform on the nanoscale. Despite their technological importance, the real space details have remained elusive. Here we reveal and study nanoscale crosslink heterogeneities in colloidal gel particles that have been predicted but never actually observed in all three dimensions in real space. This opens up the possibility of tying these crucial heterogeneities to their origin during the nucleation and growth process and establishing their impact on mechanical and transport properties. Although scattering studies have provided valuable insights, the averaging inherent to scattering has obscured examining individual particles. Nascent demonstrations applying super-resolution microscopy to colloidal gels have pointed a way forward, but have not been privy to the morphologies that we now study. Super-resolution imaging of spatial heterogeneities in bulk hydrogels, which we also demonstrate, have not even been attempted. The additional insight our work brings to materials science relates to the incidence, nature and origin of fractal or self-similar materials.

## Introduction

Most polymer networks, and gels in particular, possess variations in crosslink density.^1^ In gels, these so-called spatial heterogeneities manifest themselves on the scale of tens of nanometers, and are of significant scientific and technological interest as they directly impact the mechanical and transport properties of the gel. This is particularly true of colloidal gel particles, or microgels, which exhibit high surface area per unit mass and swift swelling equilibration,^2^ and are thus candidate next generation materials in sensing,^3^ actuation^4^ and drug delivery.^5^ Despite this emphasis, the internal nanostructure is described by leading researchers as being unresolved.^6^ Valuable insight has come from scattering studies.^7^–^11^ With the circumvention of the diffraction barrier in far-field fluorescence microscopy,^12^–^14^ an exciting recent advance has been the addition of optical microscopy to the toolbox of methods that can reveal real space information in microgels^15^–^18^ and soft condensed matter in general.^19^,^20^ However, spatial heterogeneities in crosslink density have remained obscured, presumably due to insufficient resolution in the third dimension or the assumption of spherical symmetry while extracting structural information.

Here we reveal the real space morphology of spatial heterogeneities in a model crosslink labelled poly(*N*-isopropylacrylamide) (PNiPAm) microgel system using state of the art two lens super-resolution microscopy called Whole cell 4Pi Single Molecule Switching Nanoscopy (W-4PiSMSN).^21^ By introducing dilute quantities of a novel dye tagged hydrophilic crosslinker we undertake direct *in situ* 3D imaging of crosslink distributions within the microgels with W-4PiSMSN, which has a demonstrated 10–20 nm isotropic resolution even in aberration inducing samples that are ten microns thick. We find that the core of individual microgels consists of clusters of higher crosslink density on the order of several tens of nanometers, embedded in a matrix of lower crosslink density. We examine the size distribution of the clusters, and find that the individual clusters themselves are inhomogeneous in a manner that deviates from the classic fuzzy sphere model for microgels predicted by scattering studies.^8^ Such deviations have recently been seen for the polymer volume fraction of highly crosslinked microgels.^18^ Finally, we find that the ensemble averaged radial dye tagged crosslink density distribution is consistent with the fuzzy sphere model of the polymer volume fraction distribution. Taken together, this serves as compelling evidence for a previously proposed model for the early stages of microgel formation by precipitation polymerization, which in turn explains the nature of the ensemble averaged radial polymer volume fraction variation.^2^,^22^ The size distribution, internal morphology and shape of the high density clusters provides insight into the nature of the nucleation and growth of microgels.

## Results and discussion

**Fig. 1:**
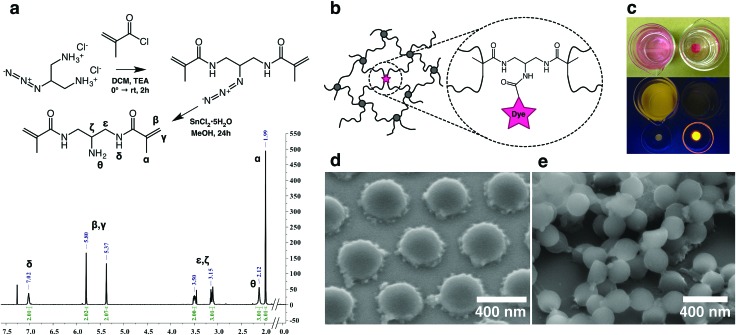
(a) Synthetic route and ^1^H-NMR spectrum of BMA-NH_2_. (b) Representation of hydrogel mesh with dye tagged crosslinks. (c) Top frame: appearance of physically infused (left) and covalently-tagged hydrogels (right) after immersion in water overnight. Bottom frame: hydrogels and their supernatants illuminated by UV-light. (d) SEM micrograph of dried microgel particles displaying morphologies typical of spatial crosslink density variations. (e) SEM micrograph of flash dried microgel particles. The top layer corroborates the spherical nature of the particles.

To facilitate imaging with W-4PiSMSN we first synthesized a novel amine functional hydrophilic methacrylamide crosslinker. The guidelines for the design of the crosslinker were set as follows: (i) molecular dimensions and composition similar to the common crosslinker *N*,*N*′-methylenebis(acrylamide) (BIS) (ii) water solubility and compatibility with free radical polymerization reactions in aqueous media and (iii) highly reactive pendant functionality. The synthetic scheme (Scheme S1, ESI†) was inspired by the work of Moszner *et al.*^23^,^24^ on hard resin adhesives. Fig. 1a summarizes the synthesis of the target compound *N*,*N*′-(2-aminopropane-1,3-diyl)bis(2-methylacrylamide) (BMA-NH_2_), with 2-azidopropane-1,3-diaminium chloride,^25^ as the starting point. Dye tagged crosslinkers were obtained by reacting the BMA-NH_2_ with activated ester derivatives of either Rhodamine or ALEXA 647 to yield BMA-Rh and BMA-ALX, respectively. Covalent incorporation was verified by using BMA-Rh (Fig. 1b) in the synthesis of PNiPAm hydrogels and subsequent immersion of the hydrogel stub in water. Hydrogel samples containing physically trapped Rhodamine served as a negative control (Fig. 1c). A negligible amount of residual unconjugated ester derivative and unreacted BMA-Rh accounts for the minor dye leaching observed in the covalently bonded case shortly after immersion. In stark contrast, free dye diffuses continuously from the control hydrogel matrix in the experimental timeframe (Video S1, ESI†).

Next, tagged microgels (MGPNM@ALX) were synthesized using 2% BIS and 0.01% functional crosslinker (containing BMA-ALX and BMA-NH_2_), molar eq. relative to NiPAm (see Table S1 in ESI† for amounts). Using variable temperature dynamic light scattering the resultant particles were found to be monodisperse with sizes of *D*_h,20°C_ = 666 nm and *D*_h,40°C_ = 305 nm in the swollen and deswollen state, respectively (Fig. S1, ESI†). Scanning electron microscopy of drop cast particles (Fig. 1d) captures the presence of a flower petal like corona typical of microgels that possess a higher crosslinked core with a radially diminishing crosslinking density. BIS is consumed faster with respect to NiPAm during polymerization^26^ and it has been shown through scattering and microscopy that the ‘petals’ are aggregates of the retracted loosely crosslinked mobile chains.^27^–^29^ Flash dried samples corroborated the spherical nature of the microgels (Fig. 1e).

**Fig. 2:**
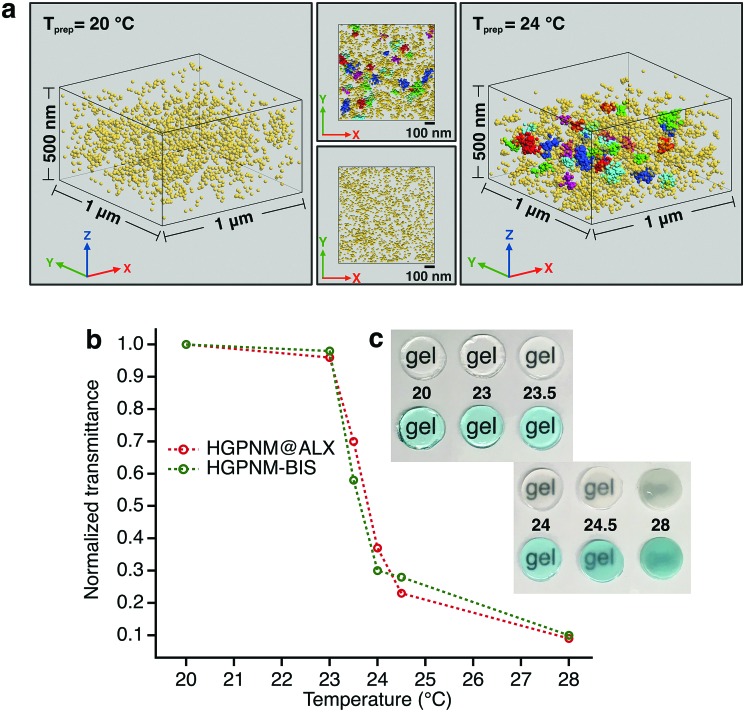
(a) Isometric representation of localized emission centers within crosslinker dye-tagged hydrogels featuring relative crosslink homogeneity (*T*_prep_ = 20 °C) and nanoscale spatial crosslink heterogeneities (*T*_prep_ = 24 °C) (b) normalized transmittance of dye-tagged and pristine hydrogels featuring different degrees of heterogeneity (c) optical appearance of prepared hydrogels.

To exemplify our ability to image nanoscale variations in crosslink density in our materials system, we first imaged PNiPAm hydrogel films containing the same ratio of BIS to BMA@ALX as the microgels, with preparation temperatures of 20 °C and 24 °C, respectively (Fig. 2a). It is well established that PNiPAm hydrogels contain an increased incidence and size of spatial heterogeneities in crosslink density as their temperature of preparation is increased,^30^ as can be seen in the transparency curve shown in Fig. 2b. The dye tagged hydrogels show a transparency dependence very similar to the untagged hydrogels. To identify regions with higher crosslink density we used a density based clustering algorithm known as DBSCAN,^31^ which, given a minimum number of points per cluster and a search radius value, can identify clusters of varying size, shape and unknown number within a data set. We set a conservative search radius value of larger than twice the highest localization precision values. For films prepared at 24 °C, strong clustering was detected at length scales above the search radius, while the samples prepared at 20 °C exhibit relatively homogeneous crosslink distributions. We note that these are, to the best of our knowledge, the first super-resolution images of spatial heterogeneities in bulk hydrogels.

**Fig. 3:**
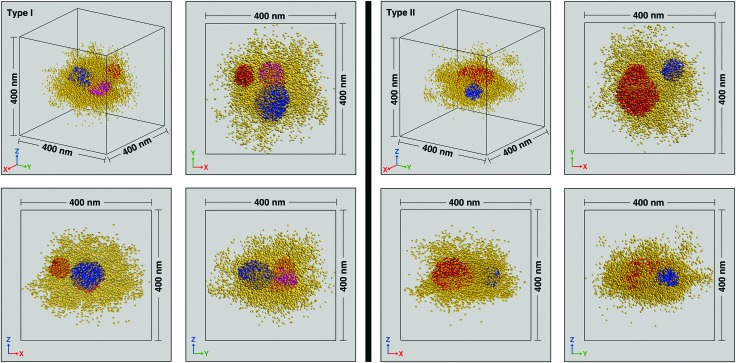
Isometric representations and *XY*, *YZ*, *XZ* projections of localized emission centers within two typical crosslinker dye-tagged individual particles featuring lower (Type I) and higher (Type II) deformation. Localizations within the higher crosslink density patches (red, blue and magenta) are plotted with higher opacity than the surrounding matrix (yellow).

Next, we probed the distribution of dye tagged crosslinks within individual swollen PNiPAm microgel particles. *XY*, *YZ* and *ZX* projections of the localized emission centers of the dye tagged crosslinkers within representative isolated particles are shown in Fig. 3. Based on the envelope of the localizations we find that the particles on the cover slip surface are oblate spheres. For simplicity, further analysis is only presented for microgels that are not significantly oblate. We defined significantly distorted spheres as those that have an aspect ratio greater than 1.5, where the aspect ratio is calculated as the ratio of the full width half maximum along the *x* and *z* directions of the histogram that results from the projection of the localizations onto the *xy* and *xz* planes respectively. Particles with aspect ratios less and greater than 1.5 are labeled Type I and Type II, respectively. The use of two coherently opposed objectives significantly improves resolution along the optical axis. Resolution in single molecule localization microscopies is also a function of the imaging conditions and the materials system. For the microgel samples reported here we calculated^32^ resolutions of 24 nm, 27 nm and 18 nm in the *x*, *y* and *z* directions, respectively (see ESI† for details).

**Fig. 4:**
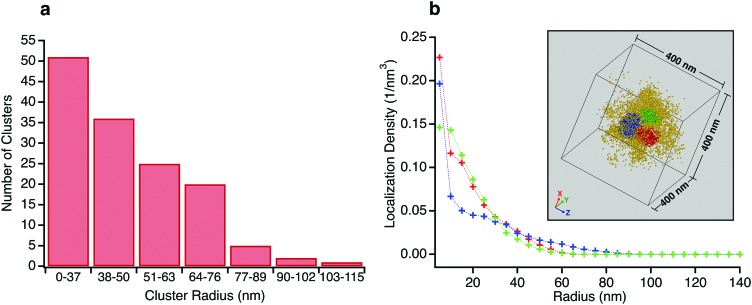
(a) Histogram of the number of clusters *versus* cluster radius for clusters recorded in Type I particles. (b) Localization probability density as a function of radius for three clusters from the same microgel (shown in inset). The densities in the regions representing the cores of the clusters are not constant.

At the individual particle level, localizations within a central region of radius ∼100 nm reveal regions of relatively higher crosslink density with extents on the scale of several tens of nanometers. There are anywhere between 1–4 clusters per microgel, with a typical number being 2 or 3. While the individual clusters tend towards a spherical envelope, they do not have a very definite interface. The clusters vary from being isolated to being almost merged, with the largest clusters (∼3% of the total) displaying peanut like morphologies (see Fig. S4 in ESI† for examples). Due to the presence of the clusters, individual particles are not spherically symmetric and the clusters do not display any preferred location within the 100 nm radius region in which they are typically found. A histogram of the number of clusters for sizes beyond the search radius value decreases monotonically (Fig. 4a). Such size distribution profiles provide an insight into the kinetics of the nucleation and growth of the microgels formed by precipitation polymerization. The clusters themselves also have a non-uniform crosslink density distribution and the localization probability density within the core region of the clusters are often not constant (see Fig. 4b for an example). Such deviations from the fuzzy sphere model have been recently observed for the polymer volume fraction profile of highly crosslinked microgel particles (>5% crosslinker content) obtained *via* super-resolution microscopy, where the profile has been fit to an error function modified with a linear term.^18^ Thus, the majority of the clusters are likely to be highly crosslinked. This nanoscale heterogeneity, which is only discernible when individual immobile particles are probed with high 3D resolutions is significant as it provides compelling supporting evidence for an existing model for the formation of microgel particles.^22^ In this model, the early stages of precipitation polymerization are characterized by the formation of precursor nuclei by the collapse of polymer chains above a critical length. The heterogeneities and shapes observed are consistent with the prediction that these precursor particles subsequently aggregate, deposit on existing particles, and grow by addition of monomers, finally resulting in the “hairy” morphology seen at temperatures below the volume phase transition temperature of PNiPAm. The prediction of this model that clusters can aggregate or deposit onto clusters that have already grown by addition of monomers implies that the clusters need not be located at the center of any given microgel.

**Fig. 5:**
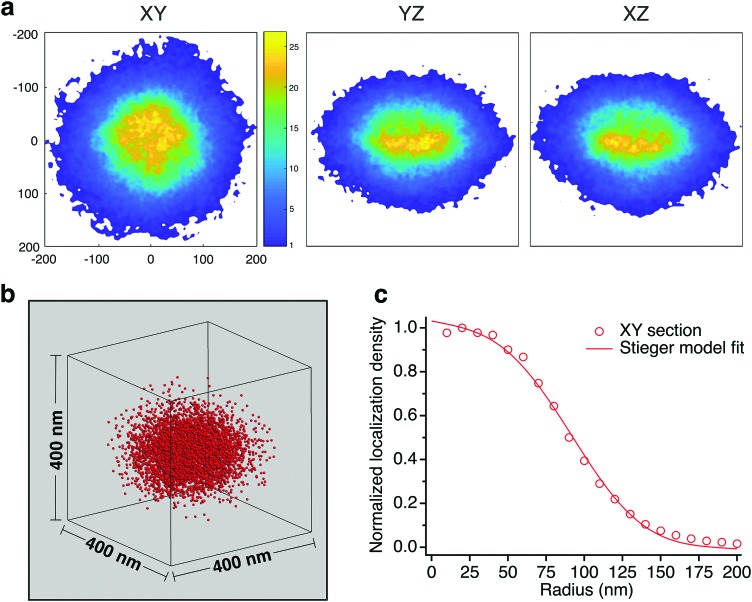
(a) Probability distribution color maps of dye-tagged crosslinks in 30 nm thick slices. (b) Isometric representation of Type I particle average. (c) Normalized localization density profile for the *XY* slice shows the characteristics of the fuzzy sphere model, which we fit to the Stieger model.

On average, the distribution of the dye tagged crosslinker shows a higher concentration at the core which diminishes beyond a certain radius. Fig. 5 shows color maps of the probability distribution of BMA@ALX averaged over Type I particles for slices with 30 nm thickness in the *XY*, *YZ* and *XZ* planes (Methods, ESI†). We reduced the data to a 1-D plot of probability density across 10 nm thick shells as a function of radial distance, using the *XY* slice as it reflects more closely the sphericity of the particles in suspension. The shape matches the distribution of polymer volume fraction seen in ensemble averaged scattering experiments.^8^,^11^ A fit to the Stieger model^8^ of the dye tagged crosslinker distribution yields values of *R* = 103 nm and *σ* = 34 nm. It is reasonable to assume that the crosslink density distribution of BIS also follows this profile, albeit with a later drop off, given a reactivity ratio of 
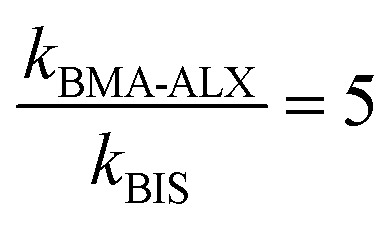
 (see Methods, ESI†), and that *R*_h_ = 333 nm.

Prior to closing, we briefly examine the possibility of the observed clusters being an artifact of multiple blinking or persistent fluorophores, as opposed to having a morphological basis. First, we consider the implications of the hydrogel images presented. It is known from scattering and transparency studies that the hydrogels prepared at 20 °C are homogeneous and the 24 °C hydrogels possess clusters on the length scales observed in the microgels.^30^ The recording of clusters only in the 24 °C samples, coupled with the fact that the localization densities of the homogenous “matrix” regions in the 24 °C sample and the homogeneous 20 °C sample are the same, is compelling evidence for the morphological basis of the clusters in this material system. Additionally, we treated localizations that appeared on consecutive frames and within 50 nm of each other as arising from persistent emission of the same fluorophore. Despite linking molecules over spherical volumes with diameters nearly ten times the typical Cramer–Rao lower bounds, we find that the number of clusters remains the same and the sizes change less than 10%, with increases in some cases and decreases in others (see Fig. S7 for representative examples, ESI†). Finally, we evaluated the potential for multiple blinking being the cause of the clusters. Annibale *et al.*^33^ have shown that clusters arising from multiple blinking artifacts are associated with concomitant spatial and temporal clustering, which can be assessed from cluster kymographs (see Fig. S8 (ESI†) for representative kymographs of clusters from the microgels and the heterogeneous 24 °C hydrogel). The temporal distribution of the microgel cluster kymographs compare favorably with the temporal distribution of localizations that Annibale *et al.*^33^ associate with the absence of multiblinking induced clustering artifacts. Furthermore, the temporal distribution of microgel clusters also compares favorably with those of the clusters of the 24 °C hydrogels, the existence of which have been unambiguously established by transparency and SANS studies,^30^ as well as the relatively lower dye tagged crosslinker density regions in which the clusters are embedded.

In summary, we have imaged the spatial distribution of dye tagged hydrophilic methacrylamide crosslink points in p(NIPAM-*co*-BIS) copolymer microgels. Examination of individual microgels reveals that the core consists of higher crosslink regions embedded in a lower crosslink matrix. We observe strong hints of a fractal, self-similar structure; the clusters show a radial crosslink density profile that decreases radially, while the microgels on average show a profile consistent with the predictions for polymer volume fraction seen in scattering studies. This provides real space evidence for a predicted model of the nucleation and growth of such microgels. This study also serves as proof-of-concept of the high resolution 3-D real-space mapping of crosslink density variations in bulk hydrogels. Given the importance of spatial distributions of crosslinks in determining the structure property relations of microgels, and gels in general, we anticipate that these results will augment the rational design of particle systems for tailored applications and the systematic study of the impact of spatial heterogeneities in bulk hydrogels.

## Funding sources

This work is based in part upon work supported by the National Science Foundation under grant no. 1654599. Acknowledgement is made to the Donors of the American Chemical Society Petroleum Research Fund for partial support of this research. This work is based in part upon work supported by the Wellcome Trust (203285/B/16/Z) and the Yale Diabetes Research Center (NIH P30 DK045735).

## Conflicts of interest

J. B. discloses significant financial interest in Bruker Corp. and Hamamatsu Photonics. The remaining authors have no conflicts to declare.

## Supplementary Material

Supplementary information

Supplementary movie
