# Supplementary material for: 3D mapping of nanoscale crosslink heterogeneities in microgels
Source: Mater Horiz. 2018 Sep 5;5(6):1130–6. doi: 10.1039/c8mh00644j (PMC6208052; doi:10.1039/c8mh00644j)
Supplement: Supplementary file 1 [file MH-005-C8MH00644J-s001.pdf]

Electronic Supplementary Material (ESI) for Materials Horizons.
This journal is © The Royal Society of Chemistry 2018

Supporting Information

3D Mapping of Nanoscale Crosslink Heterogeneities in Microgels

Apostolos A. Karanastasis[†], *Yongdeng Zhang*[‡], *Gopal S. Kenath*[†], *Mark D. Lessard*[‡], *Joerg Bewersdorf*[‡,¶], *Chaitanya K. Ullal*[†*]

[†] *Department of Materials Science and Engineering, Rensselaer Polytechnic Institute, Troy, New York 12180, USA*

[‡] *Department of Cell Biology, Yale University, New Haven, CT, 06520 USA.*

[¶] *Department of Biomedical Engineering, Yale University, New Haven, CT, 06520USA*

A. Materials

1,3-Diamino-2-propanol (DAP) (Alfa Aesar, 97%), triethylamine (TEA) (Sigma, ≥99.5%), methanol (MeOH) (anhydrous, Sigma, 99.8%), Di-tert-butyl dicarbonate (Boc anhydrite) (Sigma, 99%), dichloromethane (DCM) (anhydrous, Sigma, 99.8%), methanesulfonyl chloride (MsCl) (Sigma, ≥99.7%), *N,N*-dimethylformamide (DMF) (anhydrous, Sigma, 99.8%), tin(II) chloride dihydrate ($SnCl_2{\bullet}2H_2O$) (Sigma, 98%), 2,6-di-tert-butyl-4-methylphenol (BHT) (Sigma, 99%), Alexa Fluor 647 NHS Ester (ALX-NHS) (Molecular Probes), NHS-rhodamine (Rh-NHS) (Thermo Scientific), acetonitrile (MeCN) (anhydrous, Sigma, 99.8%), Rhodamine-B (RhB) (Alfa Aesar), *N,N,N′,N′*-Tetramethylethylenediamine (TEMED) (Sigma, ≥99.9%), hexadecyltrimethylammonium bromide (CTAB) Acros Organics) were used as received. TLC plates (silica gel matrix, fluorescent indicator) were purchased from Sigma. *N*-

isopropylacrylamide (NiPAm) (Sigma, 97%) was purified twice by recrystallization from hexane. Double distilled H_2O was filtered through 0.2 um GHP syringe filter (Pall Laboratory) prior use.

B. Methods

Proton (^{1}H) NMR spectra were recorded with an Agilent 500 MHz instrument. Electrospray ionization/mass spectroscopy (ESI-MS) was carried out with a Thermo Scientific LTQ Orbitrap XL instrument. Analytical HPLC was performed on a Shimadzu Prominence system equipped with a LC-10Ai solvent delivery unit and a Photodiode Array Detector (PDA) fixed at 225 nm. A Kinetex 2.6 μm Polar C18 column was used as the stationary phase. Mobile phase consisted of a H_2O/MeOH mixture eluting on a linear gradient from 100% H_2O to 50% MeOH and a flow rate of 0.5 ml/min. HPLC peak data analysis was performed on the Igor Pro 8 suite. Time-lapse video was recorded under UV illumination using an Apple iPod touch. Microgels were purified by ultracentrifugation using a Beckman Optima LE 80K. DLS was performed with a Brookhaven 90Plus instrument operating at a 90° geometry. Samples were diluted to approximately 50 ppm and thermally equilibrated for 20 min prior to size measurements. Hydrodynamic diameter values are given as the intensity based average of 4 consecutive measurements. Samples were drop-cast on Si wafers from 0.002% w/v dispersions, dried in ambient conditions and sputtered with Pt/Au for SEM observation. 45° tilt micrographs were obtained with a Zeiss SUPRA 55 FESEM. 45° tilt and cross-sectional micrographs were obtained with a FEI Versa 3D instrument.

Dry hydrogel thin films were reconstituted in STORM buffer (containing *β*-mercaptoethanol, glucose oxidase and catalase) prior to imaging with the W-4PiSMSN system (equipped with two silicone oil immersion objectives, Olympus, NA = 1.35). Microgel samples for imaging were prepared by casting 40 ul of 0.01% w/v dispersion on polylysine coated circular glass coverslips.

The particle coverage was optimum at 15 min of deposition, after which particles not adhered to the surface were washed away by gently rinsing with ultrapure H_2O. After drying, microgels were reconstituted in STORM buffer for at least 1 h. Samples were imaged at 200 fps at a laser (642 nm) intensity about 15 kW/cm^2 and the data analysis was done as previously described[1]. Calculation of the resolution for the imaged samples and assessment of the potential impact of multiple blinking is presented in F1.

Clusters and particles were identified from the 3D localization data by using a DBSCAN algorithm. For hydrogels, the threshold size (epsilon) and number (minpts) were identified by varying epsilon and minpts until the number of clusters was maximized, while always keeping epsilon above 25nm. This resulted in epsilon and minpts values of 45 nm and 30 points respectively. Individual microgels were isolated using DBSCAN by setting epsilon and minpts at 500 nm and 1000 points respectively. XY, YZ and XZ projections of the microgels were mapped and 67 relatively non-oblate microgels identified. An ensemble microgel, comprising all the localizations from the 67 microgels, was constructed. Localizations within 30 nm thick XY, XZ & YZ slices passing through the center of the ensemble microgel were identified and each replaced with a 2D Gaussian probability distribution with a FWHM determined by the Cramer-Rao lower bound value associated with each localization. Each peak value was adjusted such that the probability under each Gaussian remained equal to 1. From the resulting localization probability map, the probability per unit area for 10 nm wide rings was plotted against distance from the center. Nanoclusters within the individual relatively non-oblate microgels were identified using the DBSCAN algorithm. The value of epsilon was set at 25 nm. For each microgel, minpts was identified as the minimum value at which the number of clusters within the microgel is maximized, while also keeping the ratio of minpts to total number of localizations in the respective microgel

within the range 0.027±0.0027 (see figure S3). For the radial localization density plot of the clusters, a 3D Gaussian probability distribution with a FWHM determined by the Cramer-Rao lower bound value was associated with each localization. Each peak value was adjusted such that the probability under each Gaussian remained equal to 1. From the resulting localization probability map, the probability per unit volume for 5 nm thick spherical shells was plotted against distance from the center.

Transmittance measurements were taken using a green LED illuminating source (ThorLabs) and power meter (Newport 2931-C). Samples were placed between the source and detector and two sets of measurements were realized after disassembling the holder and exchanging coverslips. Dark background was measured ~30 nW and 100% transmittance was set at ~70 μW.

C1. Molecular synthesis

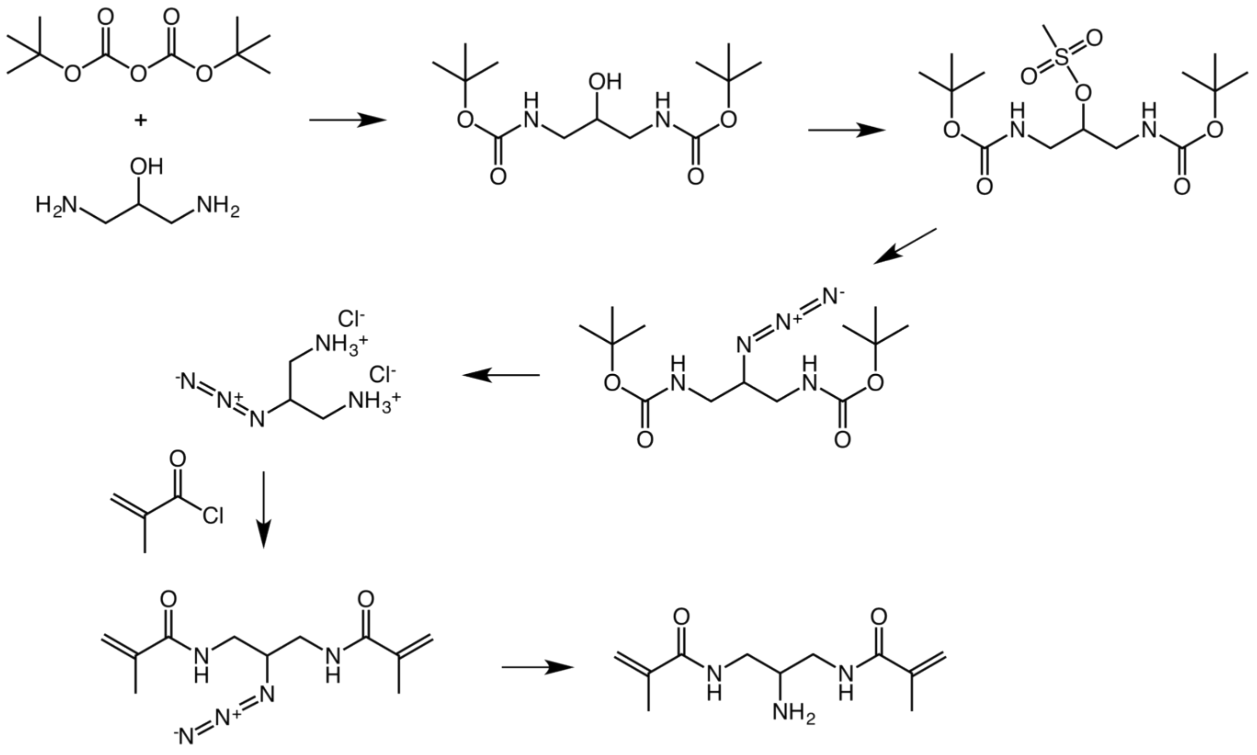

Scheme S1. BMA-NH_2 synthesis overview

N,N'-bis(di-tert-butoxycarbonyl)-1,3-diaminopropan-2-ol[2-3] (DAP-Boc)

2 g DAP (22.19 mmol), 3.14 ml TEA (22.52 mmol) and 75 ml MeOH were combined in a 250 ml round bottom flask equipped with a rubber septum and brought to 45 °C in an oil bath. 10.66 g of Boc anhydrite (48.84 mmol) were dissolved in 50 ml MeOH, loaded in an addition funnel and slowly added into the reaction under stirring. After 8 h, MeOH was rotary evaporated to afford the crude product as a yellowish slurry which was purified with silica gel flash chromatography using EtOAc/Hex (80:20) as the eluent. Solvents were removed under vacuum and the pure compound was obtained as a colorless viscous oil which solidified upon standing at -20 °C for 2 days (white solid, yield: 75%)

N,N'-bis(di-tert-butoxycarbonyl)-2-methanesulfonyl-1,3-diamino-propane[4] (DAP-Boc-OMs)

1.65 g DAP-Boc (5.69 mmol), 1.19 ml TEA (8.53 mmol) and 33 ml DCM were combined in a 100 ml rb equipped with a rubber septum. 0.461 ml of MsCl (5.96 mmol) were slowly added into the reaction under stirring at 0 °C. The reaction was left stirring for an additional 1 h at r.t., after which it attains a yellowish color indicating completion. DCM was rotary evaporated and the crude was redissolved in EtOAc. Solids were filtered out with the aid of a glass frit and the filtrate was washed with H_2O, sat. Na_2CO_3 and brine. The organic phase was dried over Na_2SO_4, filtered and concentrated under vacuum to afford the pure compound (white solid, yield: 90%)

N,N'-bis(di-tert-butoxycarbonyl)-2-azido-1,3-diaminopropane[4] (DAP-Boc-N_3)

1.8 g DAP-Boc-OMs (4.89 mmol), 1.32 g (20.31 mmol) NaN_3 and 8.5 ml DMF were combined under Ar in a 50 ml round bottom flask equipped with a septum, transferred in an oil bath at 85 °C and left to stir for 24 h. The reaction solution was poured in crushed ice (40 ml) from which an

orange pasty solid precipitated and the aqueous phase was extracted using 3×50 ml EtOAc. The crude solid was redissolved in the combined organic phases followed by washing with H_2O and brine. The organic solution was dried over Na_2SO_4, filtered and concentrated under vacuum to afford an orange oil which solidified upon standing at -20 °C for 3 days (off-white solid, yield: 81%)

2-azidopropane-1,3-diaminium chloride[5] (DAP-N_3•HCl)

CAUTION! Azide containing organic compounds with a ratio of carbon to nitrogen atoms < 1 are potentially explosives and should not be isolated in quantities more than 1 g. Always refer to relevant literature before attempting to isolate compounds with low C/N ratio[6]. 1 g DAP-Boc-N_3 (3.17 mmol) was dissolved in 9.5 ml EtOAc and 4.45 ml HCl 6M were added. The reaction was stirred overnight, after which a suspension has formed. The suspensions was cooled for 1 day and the white crystalline precipitate was thoroughly washed with EtOAc, dried under high vacuum and stored under Ar at -20 °C in a properly designated area. (white crystals, yield: 83%)

N,N'-(2-azidopropane-1,3-diyl)bis(2-methylacrylamide) (BMA-N_3)

0.32 g of DAP-N_3•HCl (1.7 mmol), 2 mg BHT and 10 ml anhydrous DCM were combined under Ar in a 50 ml round bottom flask equipped with a septum. Anhydrous TEA (2.37 ml, 17 mmol) were added to the suspension under stirring followed by 0.349 ml of methacryloyl chloride (3.57 mmol) in portions at 0 °. After 8 h at r.t the reaction was filtered through a glass frit and concentrated under vacuum. The residue was purified with silica gel flash chromatography using EtOAc as the eluent. (white solid, yield: 56%)

N,N′-(2-aminopropane-1,3-diyl)bis(2-methylacrylamide) (BMA-NH_2)

0.24 g BMA-N_3 (0.96 mmol) and 7.6 ml MeOH were combined into a 25 ml round bottom flask equipped with a septum and flushed with Ar. Under an Ar stream, 0.236 g $SnCl_2$•$2H_2O$ (1.05 mmol) were added and the reaction was left to stir. After 1h, the same amount of $SnCl_2$•$2H_2O$ was added and another 0.064 g (0.28 mmol) after 30 min. The reaction was left overnight and subsequently concentrated under vacuum, after which 20 ml DCM/TEA 90:10 were added. The resulting suspension was centrifuged and the clear supernatant was concentrated to afford an oily residue which was dissolved in DCM. The organic phase was washed 1x with 1M NaOH, dried over Na_2SO_4, concentrated under vacuum and purified with silica gel flash chromatography (DCM/MeOH 85:15, 0.2% TEA). The colorless oil which was obtained crystalized upon standing at 5 °C for 1 day (yellowish waxy crystalline solid, yield: 31%)

BMA-NH_2 conjugation reactions with dyes.

The coupling reactions was optimized at 2.5x excess of the amine, ensuring near-quantitative dye consumption and efficient use of the costly ALEXA-647.

BMA-Rh: 1 mg Rh-NHS (1.89 umol), 10 ul TEA and 1.07 mg BMA-NH_2 (4.75 umol, 1.07 ml of 1 mg/ml stock) were combined under a gentle Ar stream. The reaction was monitored with TLC using DCM/MeOH 85:15 as the eluent and near-complete consumption of the starting material was evident after 2 h. Solvents were removed in reduced pressure and the solid residue was dissolved in 1 ml H_2O to afford BMA-Rh stock solution which was kept at 5 °C in the dark until further use.

BMA-ALX: 0.4 mg ALX-NHS (0.31 umol, 400 ul of 1 mg/ml stock solution in MeCN), 10 ul TEA and 0.173 mg of BMA-NH_2 (0.77 umol, 173 ul of 1 mg/ml stock solution in MeCN) were

combined within a 1 dram vial under a gentle Ar stream. The reaction was kept overnight, concentrated under reduced pressure and redissolved by vortexing in 1 ml H_2O. The stock was kept at 5 °C in the dark until further use.

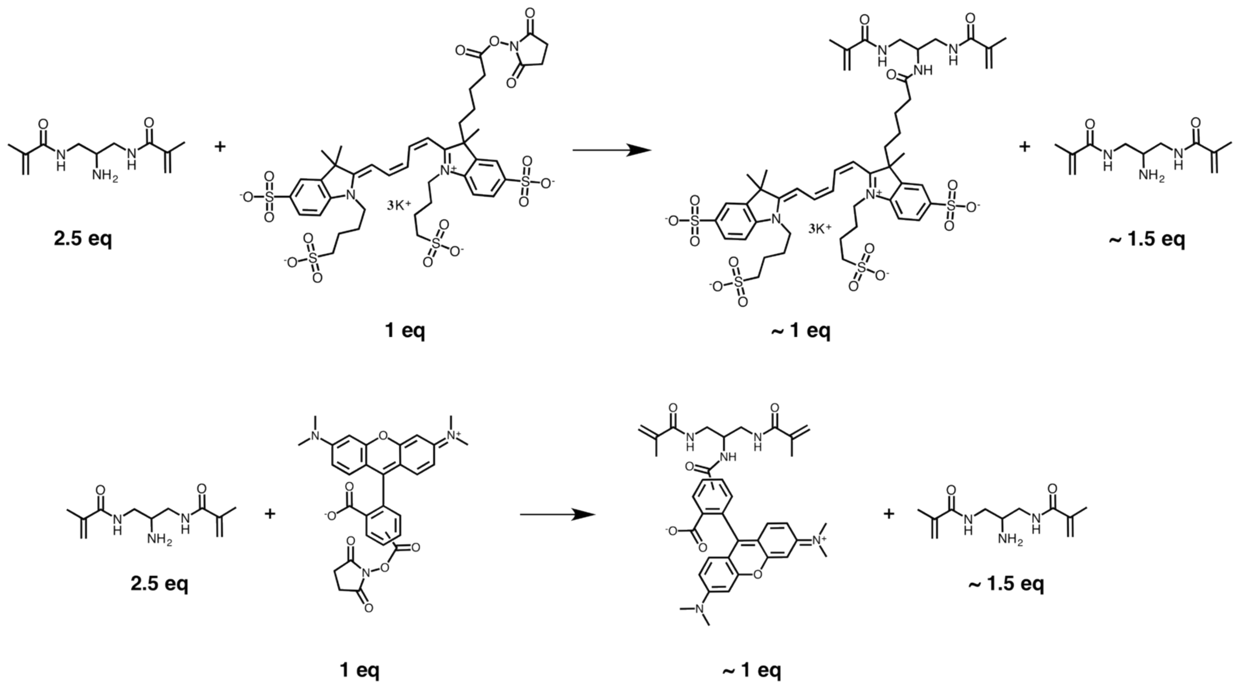

Scheme S2. BMA@ALX and BMA@Rh synthesis overview.

C2. Molecular characterization

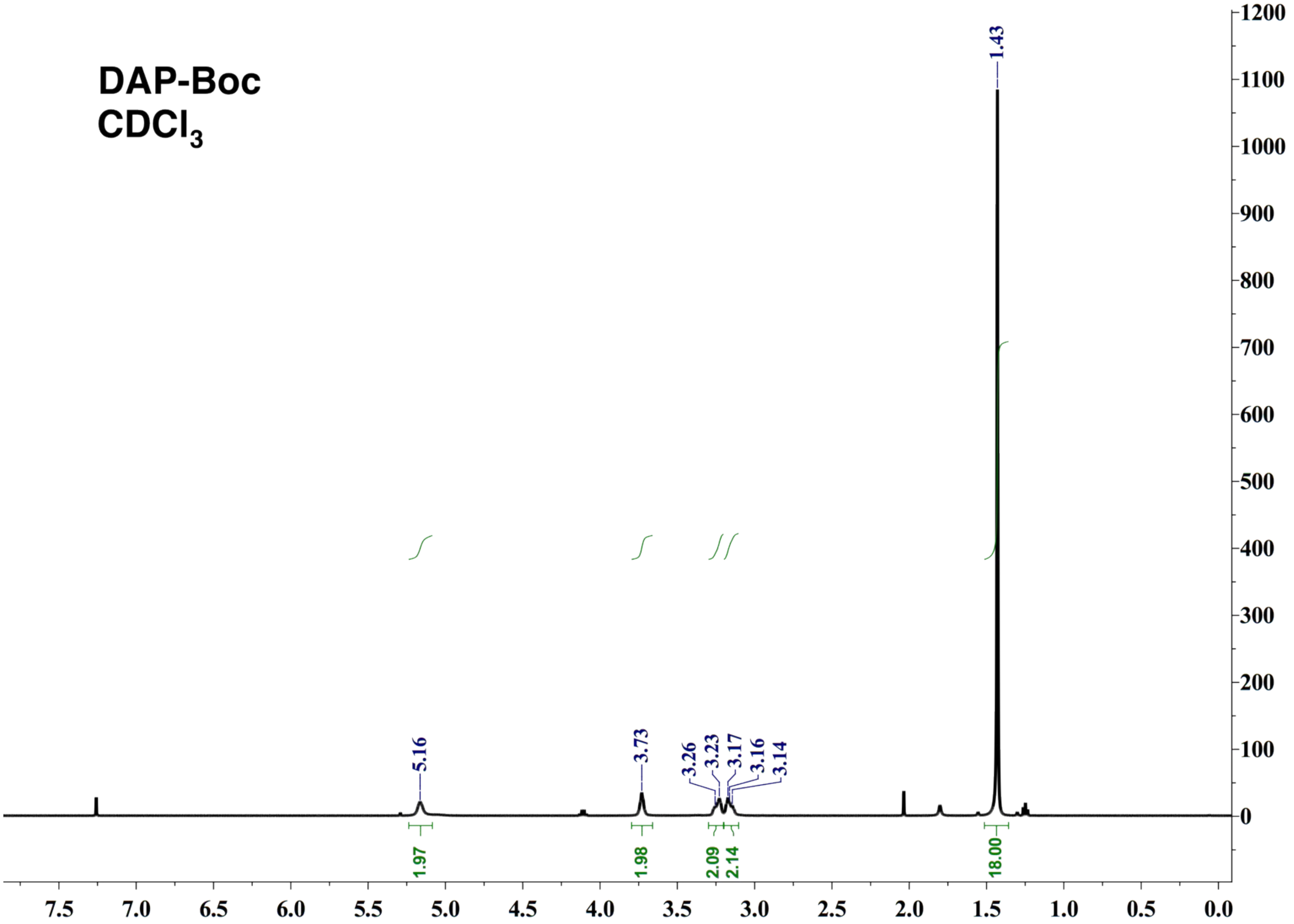

ESI-MS (DAP-Boc): 291.1935 $[M+H]^+$

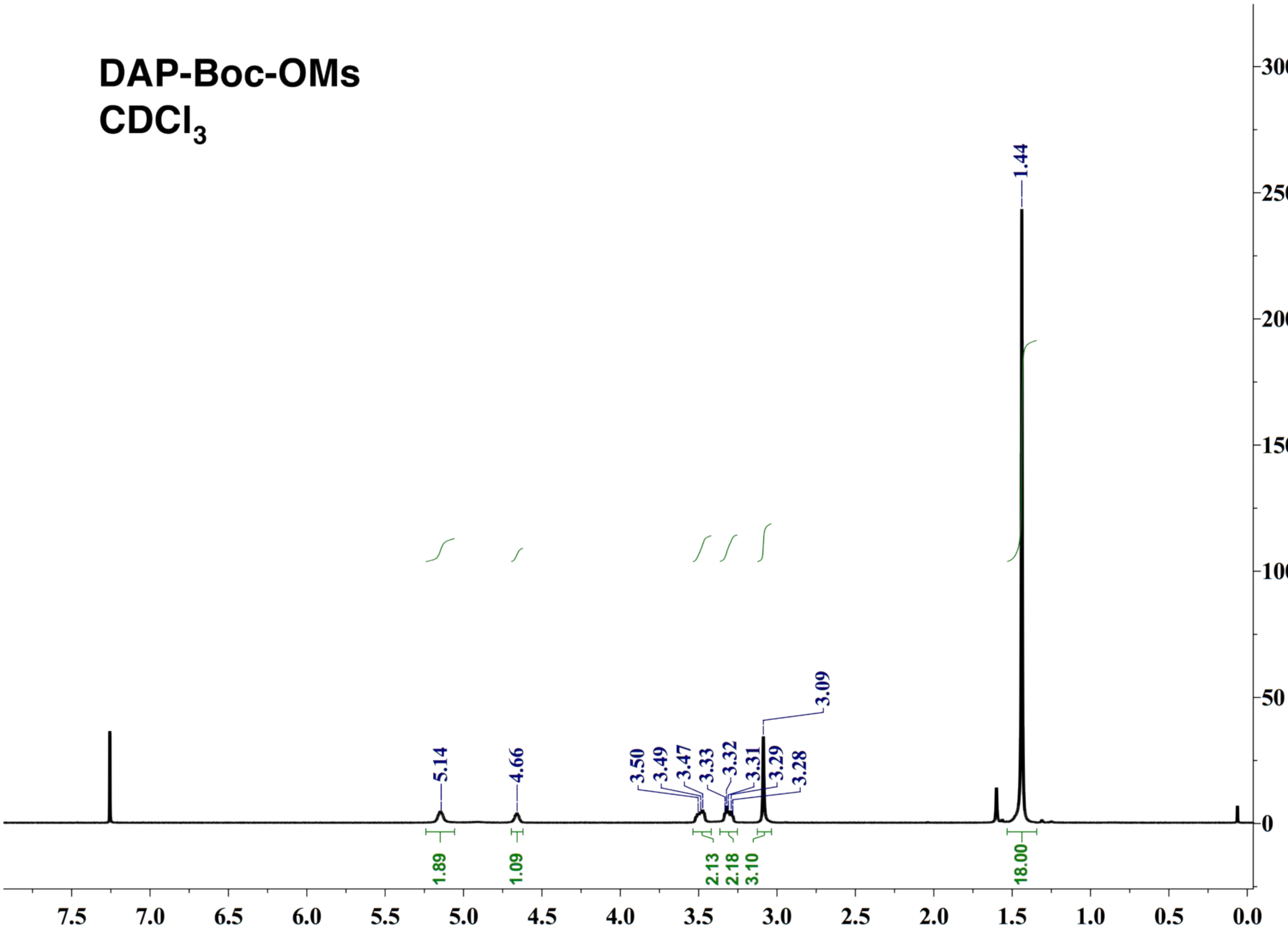

ESI-MS (DAP-Boc-OMs): 369.1705 $[M+H]^+$

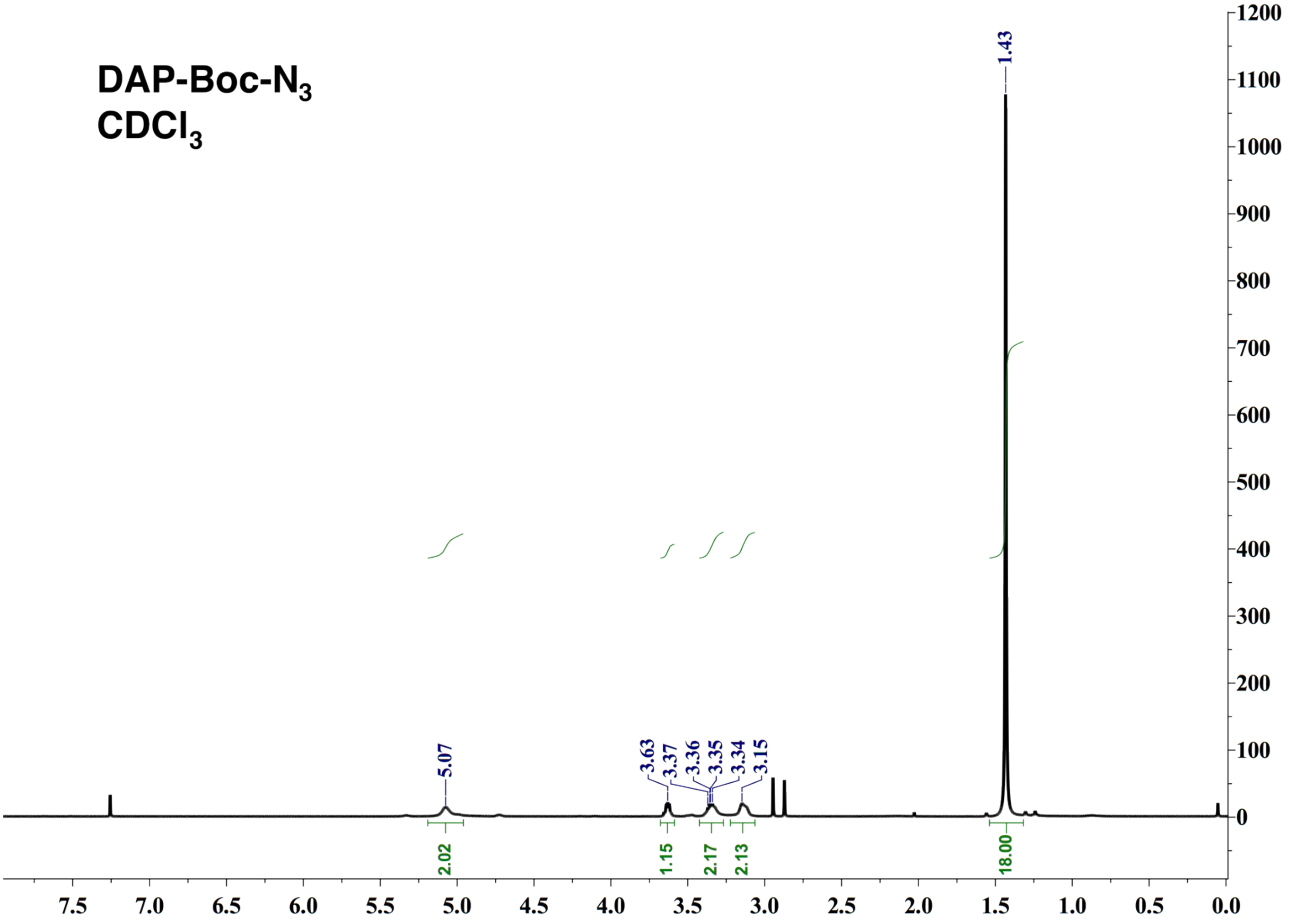

ESI-MS (DAP-Boc-N_3): 316.1988 $[M+H]^+$

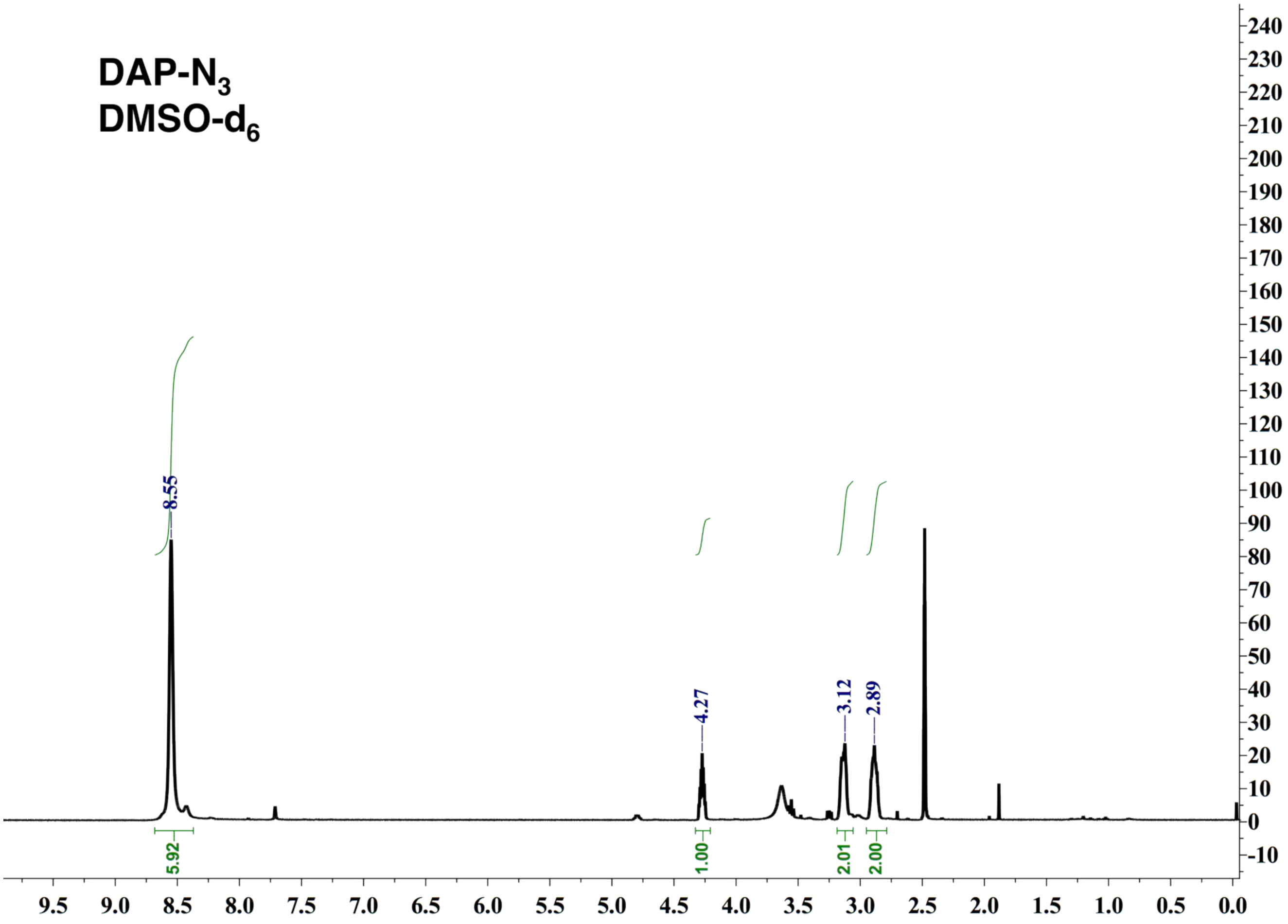
DAP-N$_3$
DMSO-d$_6$
8.55
4.27
3.12
2.89
5.92
1.00
2.01
2.00
9.5 9.0 8.5 8.0 7.5 7.0 6.5 6.0 5.5 5.0 4.5 4.0 3.5 3.0 2.5 2.0 1.5 1.0 0.5 0.0
240 230 220 210 200 190 180 170 160 150 140 130 120 110 100 90 80 70 60 50 40 30 20 10 0 -10

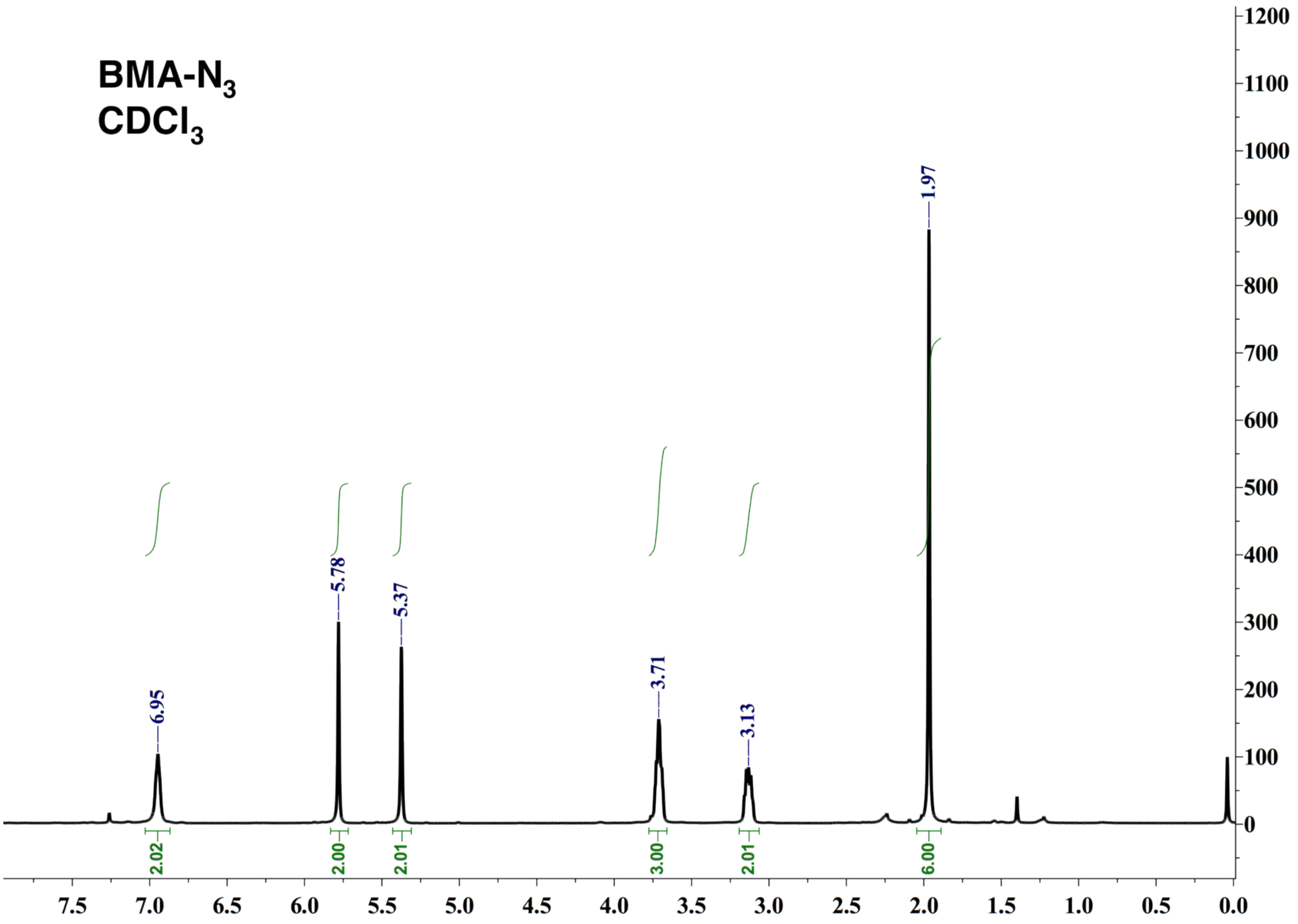

ESI-MS (BMA-N_3): 252.1463 $[M+H]^+$

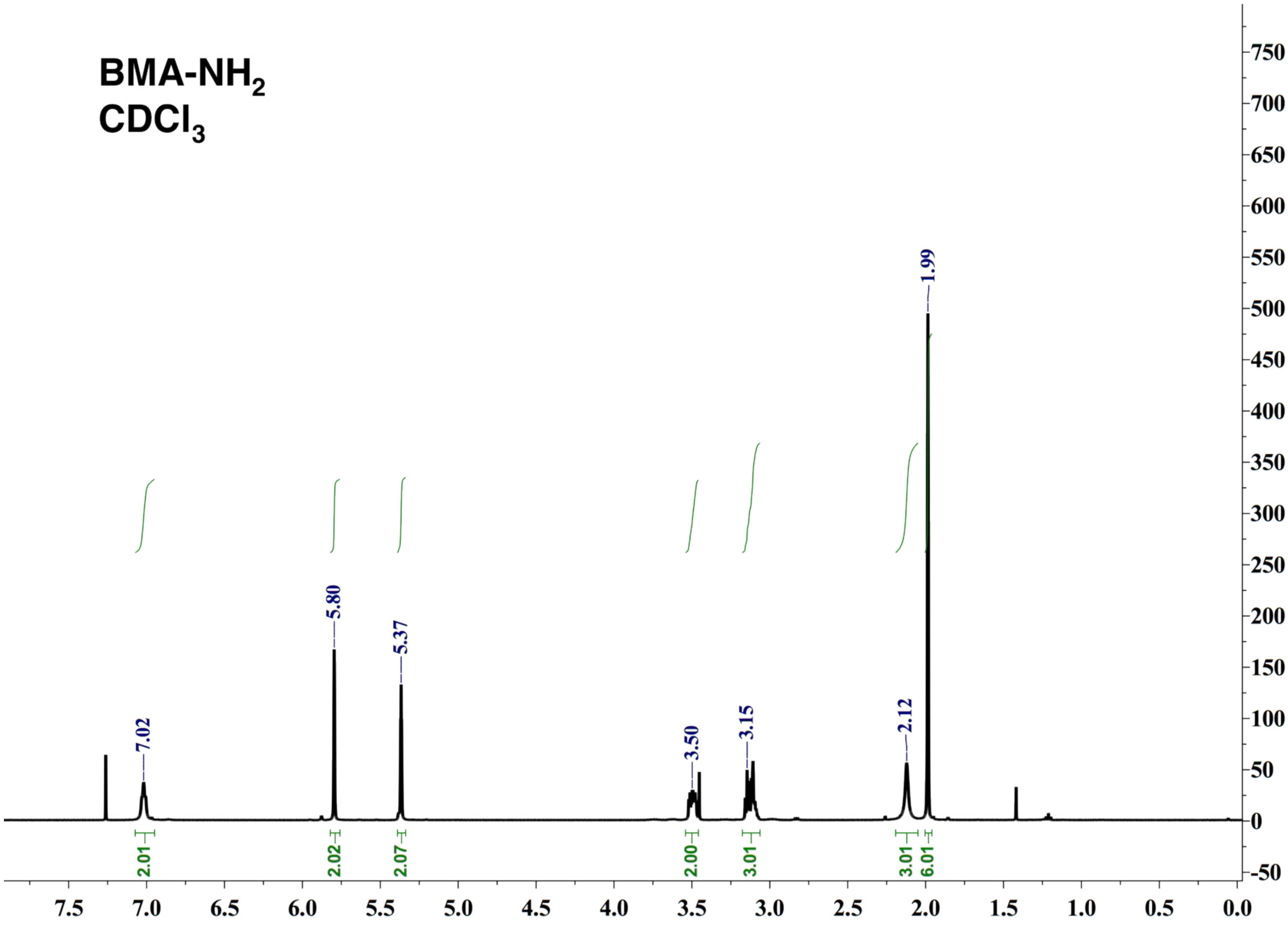

ESI-MS (BMA-NH_2): 226.1554 $[M+H]^+$

D1. Materials synthesis

Hydrogel synthesis

Covalently dye-tagged (HGPNM@Rh) and dye-infused control (HGPNM+RhB) PNiPAm hydrogels were synthesized following a protocol reported by Hashimoto et al.[7]. For the synthesis of HGPNM@Rh, 90.3 mg NiPAm (0.795 mmol), 0.56 mg BIS (3.63 umol, 30 ul of 1.85 mg/ml), 500 ul BMA-Rh dye stock and 55 ul H_2O were mixed in a 3 dram scintillation vial and cooled down to 0 °C. TEMED (3 ul) and APS (12.5 ul of 38.4 mg/ml solution) were added to the reaction solution, followed by thorough mixing, still at 0 °C. The vial was transferred in an aqueous bath at ambient temperature (~ 23 °C) for 1 h, after which the polymerization was completed. The hydrogel stub was purified by immersing 2 × 1 h in 80 ml H_2O prior recording of time-lapse. The same procedure was followed for the synthesis of the HGPNM+RhB negative control, adding 90.3 mg NiPAm (0.795 mmol), 0.925 mg BIS (6 umol, 50 ul of of 1.85 mg/ml), 0.45 mg RhB, 3 ul TEMED and 535 ul H_2O. The control stub was copiously rinsed with H_2O prior to the time-lapse video capture. For the preparation of bulk hydrogel samples for transmission measurements, the above methodology was adapted. Specifically, monomer solution handling and assembly of the polymerization cell took place in a cold room environment (~ 5 °C) in order to exclude the possibility of premature initiation at room temperature. Briefly, 150.5 mg NiPAm, 1.54 mg BIS (4.98 umol, 83 ul of 1.85 mg/ml), 63 ul of BMA-NH@ALX stock and 827 ul H_2O were mixed in a 22 ml scintillation vial, the monomer solution was cooled down to 0 °C after which TEMED (5 ul) and APS (21 ul of 38.4 mg/ml solution) were added. The monomer solution was loaded in a Presslok demountable cell holder (Sigma), using a Viton O-ring spacer (1.5 mm) for the confinement of the monomers between 25 mm circular #1 coverslips (VWR) which had been previously cleaned in triplicate with acetone, ethanol and DI water. The holder was transferred

with the aid of a cool box to a thermostated water bath at the desired temperature and left for 1 h, after which it was disassembled. The hydrogel stubs were removed, purified with frequent exchange of DI water and stored at the dark in a solvent excess. Upon reaching swelling equilibrium, the stubs were cut into 25 mm discs with the aid of a punch and mounted in the sample holder between #1 coverslips for the transmittance study. The hydrogel stubs were further cut with a 15 mm punch prior to being photographed. For ALEXA-tagged hydrogel films preparation the monomer solution was confined between two glass coverslips, following the exact same procedure as above and scaling down for 45 mg of NiPAm. Specifically, 2.5 ul of monomer solution was cast on a 25 mm circular #1.5 coverslip (Bioscience Tools) which has been thoroughly solvent cleaned. The coverslip was capped with a freshly piranha cleaned circular 25 mm #1 coverslip (VWR) and the coverslip sandwich was mounted into a Presslock sample holder. The holder assembly was transferred with the aid of a coolbox into a thermostated water bath and after 1 h the sample holder was disassembled. The coverslips where separated using a razor, exposing the laminated hydrogel film on the #1.5 solvent cleaned coverslip. The film was carefully washed with copious amounts of DI water, left to dry in ambient conditions and stored in the dark. The absence of free dye was confirmed by the absence of background in confocal images as well as the final superresolution images.

Microgel synthesis

Stable suspensions were prepared with the cationic initiator/surfactant pair V-50/CTAB. The nominal amount of free BMA-NH2 incorporated in MGPNM@ALX was 1:333 molar eq. relative to BIS, thus, the combined maximum functional crosslinker excess (BMA-NH2 + BMA-ALX) was 0.5% molar eq. relative to BIS, yielding a total crosslinker content of 2.01% molar eq. relative to NiPAm. Prior to synthesis, stock reagent solutions at prescribed concentrations were degassed

with Ar. 113 mg NiPAm (1 mmol), 3.05 mg BIS (0.02 mmol, 0.5 ml of 6.1 mg/ml), 0.24 mg CTAB (0.66 umol, 2 ml of 0.12 mg/ml), 129 ul of BMA-NH@ALX stock and 5.52 ml H_2O (V_f = 8.25 ml) were combined and degassed for 1 h in an 25 ml round bottom flask equipped with a septum and Ar gas inlet/outlet. The flask was placed in a thermostated oil bath at 80 °C for 1 h, and with the aid of a glass 500 ul airtight syringe V-50 (3.95 umol, 100 ul of 10.7 mg/ml) was injected to initiate the polymerization. The reactions were quenched in ice-water after 2 h and the crude dispersions were passed through a 1 um glass fiber syringe filter (Pall Laboratory). Samples were purified by 2 cycles of ultracentrifugation-redispersion in H_2O (at which point the supernatant is free of dye), lyophilized and stored at 5 °C in the dark. The absence of free dye was confirmed by the absence of background in confocal images as well as the final superresolution images.

Table 1. Quantities used for MGPNM@ALX synthesis**.**

	**NiPAm**	**BIS**	**CTAB**	**V-50**	**BMA@ ALX**	**BMA@ NH_2**
Mass	113 mg	3.05 mg	0.24 mg	1.07 mg	0.052 mg	0.013 mg
Molar equivalent	1 eq	0.02 eq	6.6×10^{-4} eq	3.95×10^{-3} eq	4×10^{-5} eq	6×10^{-5} eq

D2. Materials characterization

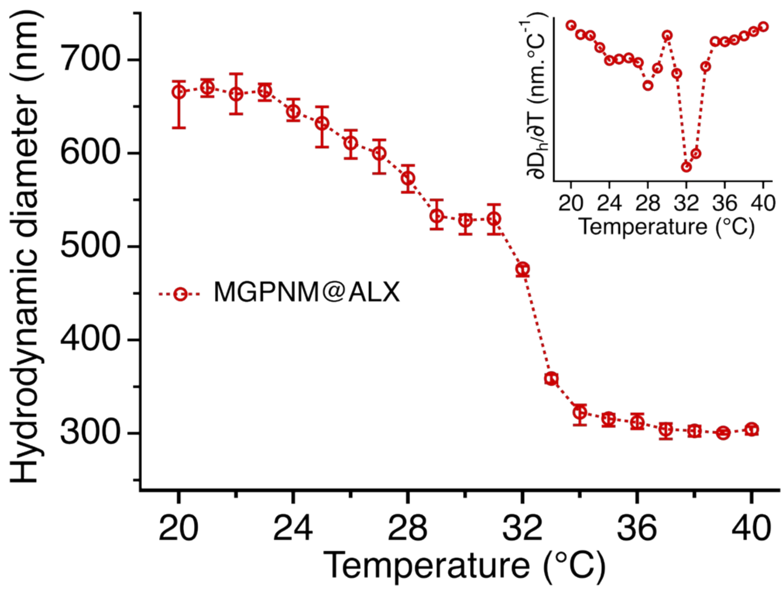

Figure S1. Variable temperature DLS of crosslink tagged MGPNM@ALX. Insets: Volume Phase Transition Temperature (VPTT) determination by differentiation of D_h in respect to T.

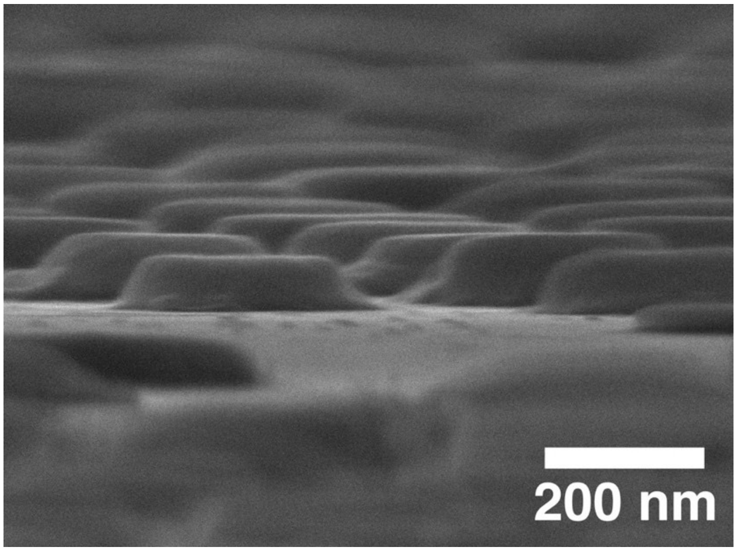

Figure S2. High magnification cross-section SEM micrographs of drop-cast MGPNM@ALX where mesa-type morphology and petal-like features, that are interpreted to be aggregates of chains, are seen at the periphery.

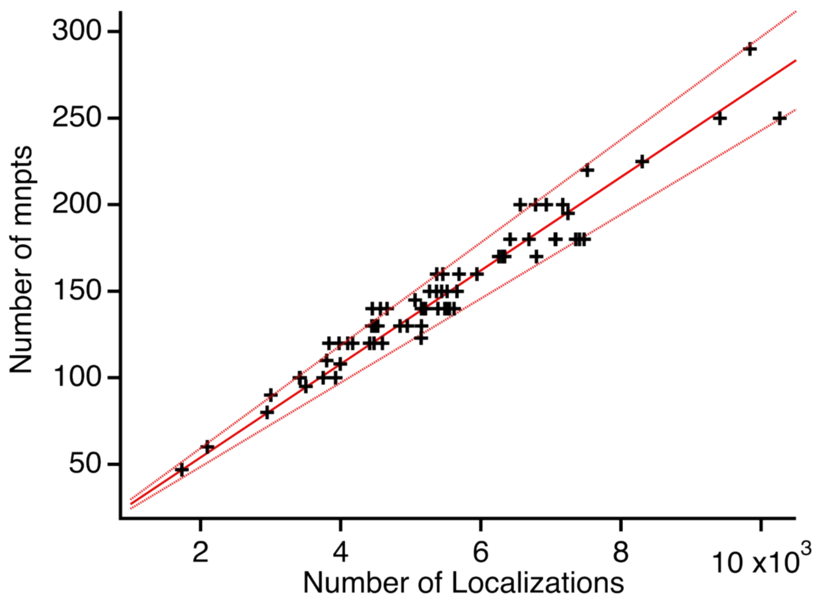

Figure S3. Number of minpts versus number of localizations scatter plots for the examined microgel dataset. Continuous line traces particles with constant ratio (0.027). Dashed lines denote boundaries within ±10% of the constant ratio.

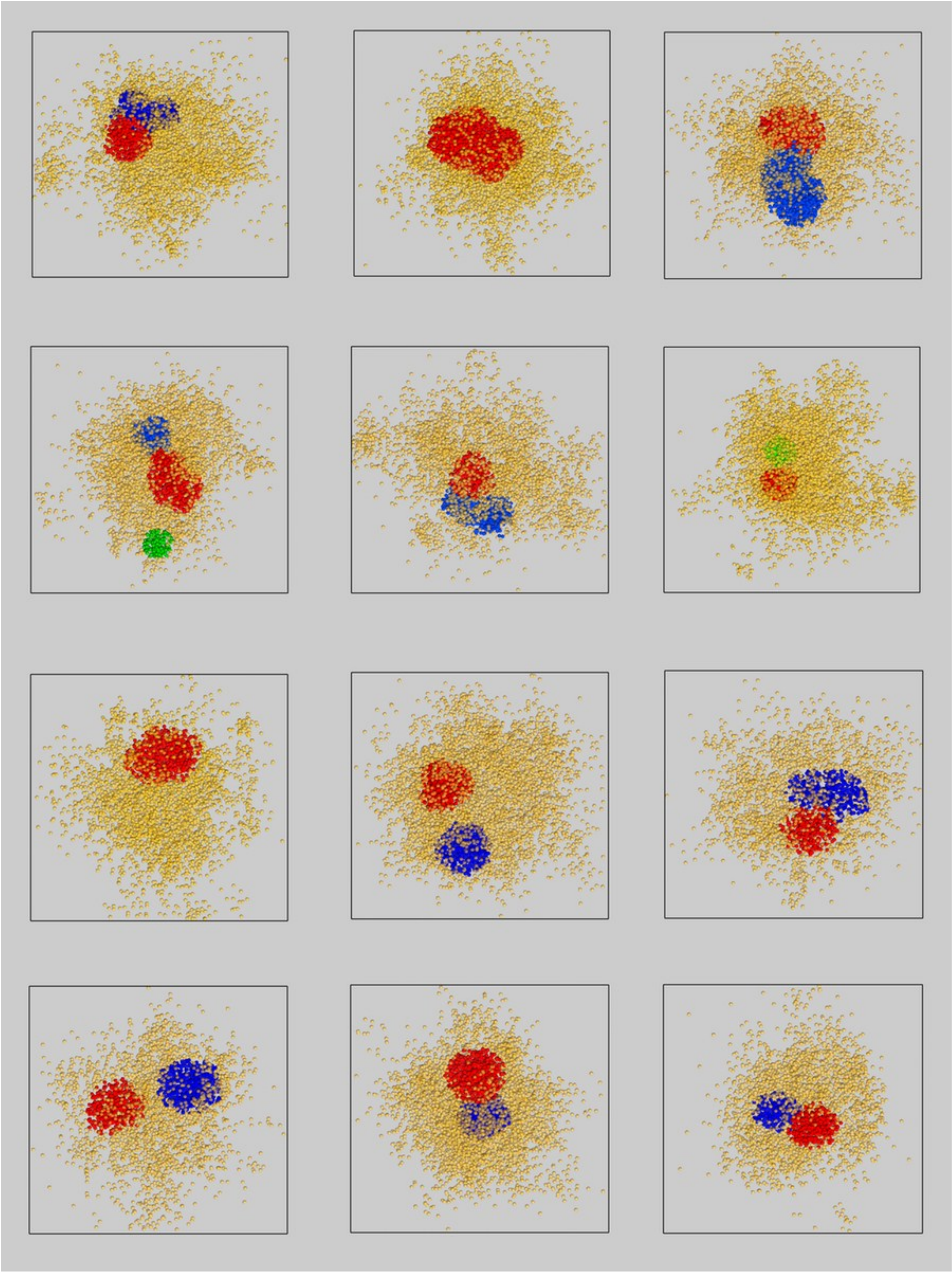

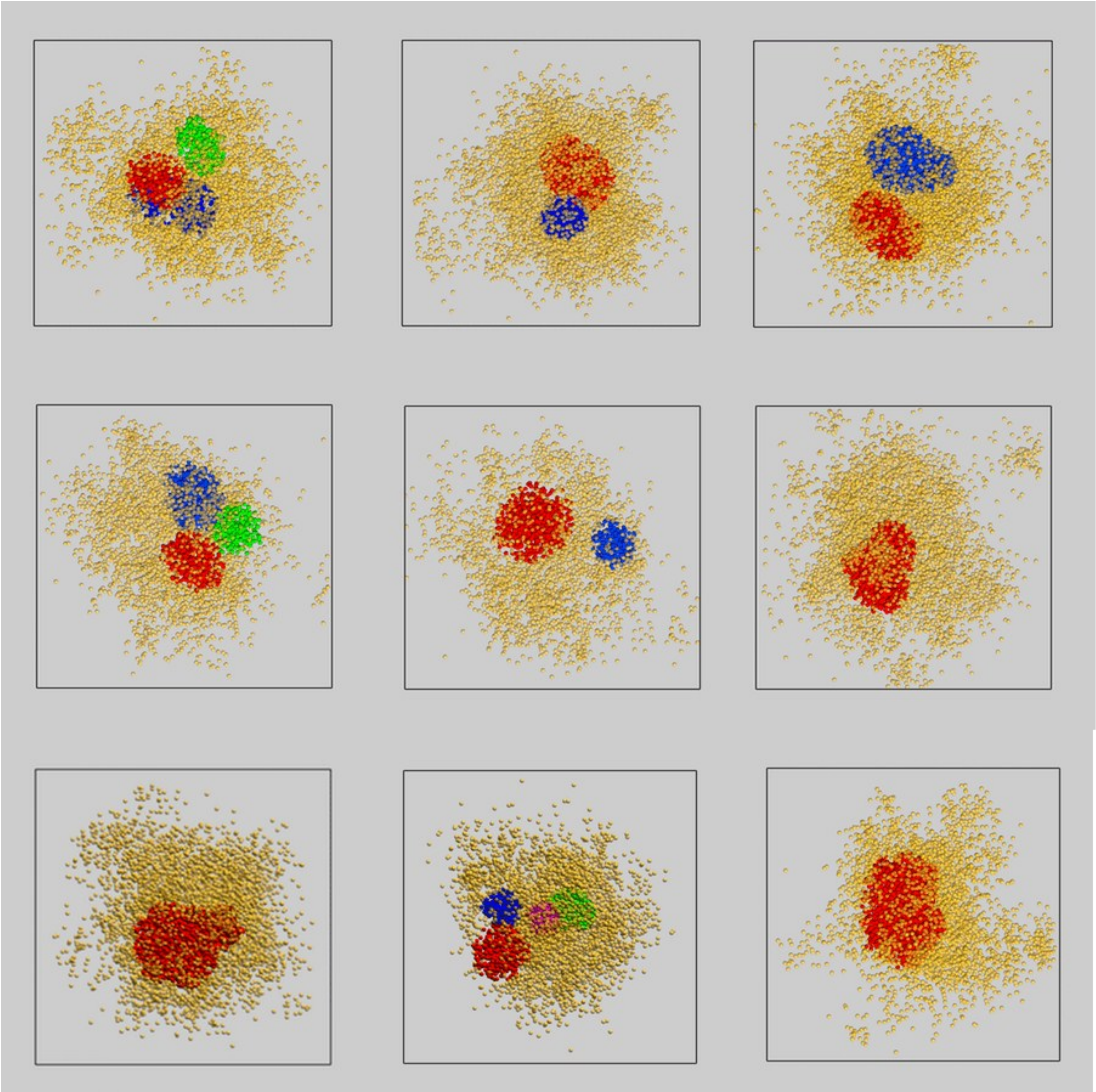

Figure S4. XY projections of localized emission centers within crosslinker dye-tagged individual particles. Localizations within the higher crosslink density clusters (red, blue, green and magenta) are plotted with higher opacity than the surrounding matrix (yellow). Edges of boxes = 400 nm.

E. Reactivity ratios of monomers.

The relative insertion kinetics were calculated by following a procedure established by Acciaro *et al.*[8] Briefly, the reaction rate of the monomers is given by the equation below:

$$\frac{dn_i}{dt} = -k_i[Pol^*][monomer_i]$$

where i = NIPAm, BIS or BMA-ALX, and Pol* denotes the polymeric radicals in the reaction mixture. The relative concentration ($C(t)/C_0$) of the monomers is plotted as a function of reaction time and the relative insertion kinetics calculated using the relationship below:

$$\frac{k_i}{k_j} = \frac{\frac{dn_i}{dt}[monomer_j]}{\frac{dn_j}{dt}[monomer_i]}$$

$C(t)/C_0$ versus time was quantified and plotted (Fig S5a) for all three monomers. $C(t)/C_0$ for NiPAm and BIS was measured by HPLC as per the procedure described in Reference 8. The ensemble averaged $C(t)/C_0$ for BMA-ALX was calculated from the dye distribution data of Fig 5, by using the size dependence of the particles on reaction time (see Fig S5b) to convert the radial dependence into the necessary time dependence. The conversion of monomer during the synthesis of microgels is consistent with the literature[8,9]. The reactivity ratio was calculated to be

$$\frac{k_{BMA@ALX}}{k_{BIS}} = 5$$

.

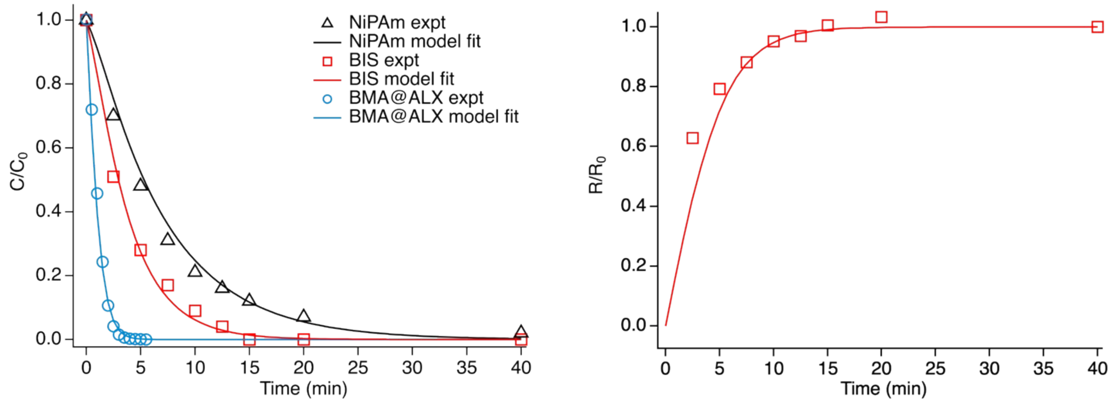

Figure S5. (a) Relative concentration ($C(t)/C_0$) of the monomers is plotted as a function of reaction time. (b) Size dependence of the particles on reaction time.

F. Resolution calculation

The resolution for the specific microgel system reported in this manuscript was calculated using the method used in Ref. 10 and 11. In brief, molecules that appear on consecutive frames and within 50 nm (in 3D) of each other are treated as arising from persistent emission of the same fluorophore and linked together. Localizations associated with molecules that are persistent across > 9 frames were identified and aligned by their center of mass to generate the localization distributions shown in Figure S6, below. From this data, we estimated the resolution to be ~24 nm in x, ~27 nm in y and ~18 nm in z.

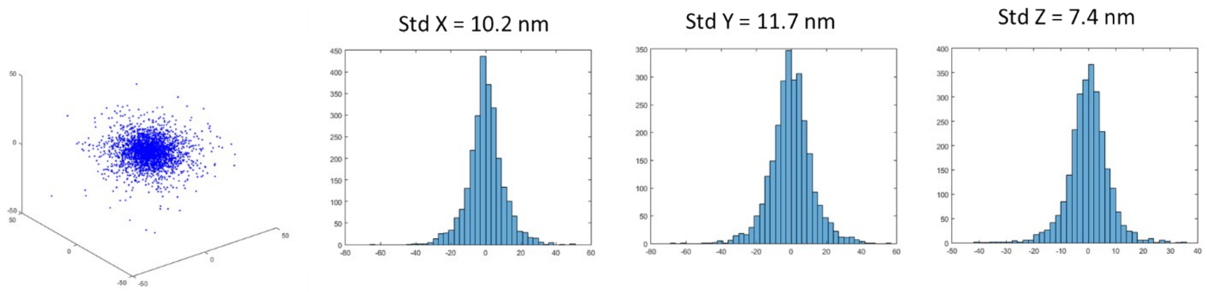

Figure S6. Resolution calculation as per Ref. 10 and 11.

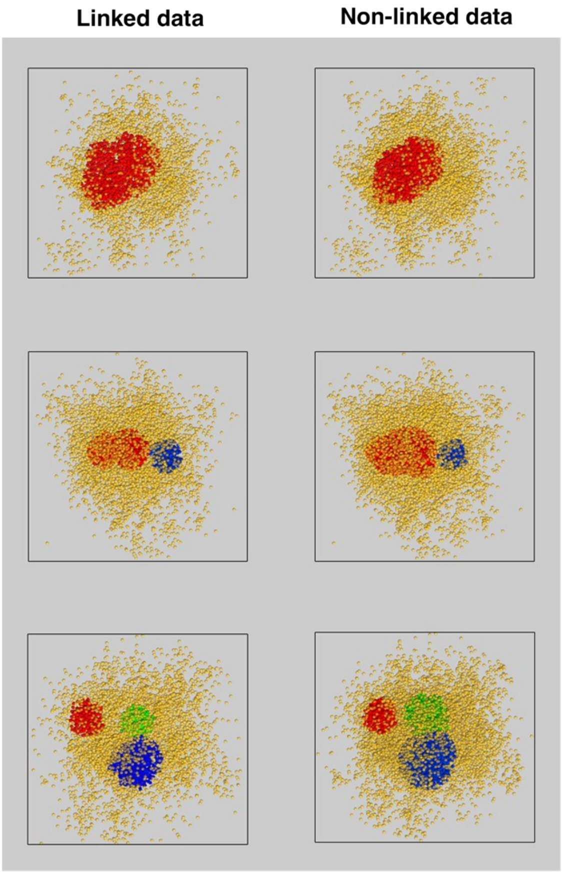

Figure S7. Representative examples of impact of "linking" localizations to identify persistent molecules on the incidence of clusters in microgels. Edges of boxes = 400 nm.

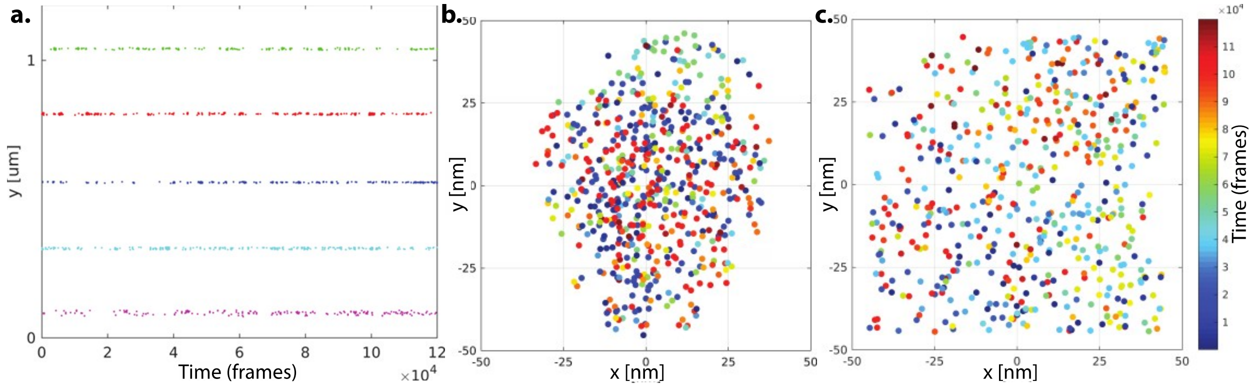

Figure S8. (a) Kymographs of (i) 3 representative clusters from different microgels (green, red and blue traces) (ii) 100 nm x 100 nm area of a microgel that does not contain clusters (cyan trace) and (iii) a cluster from the 24°C hydrogel (magenta trace). Such temporal distributions are associated with the absence of multiblinking induced clustering artifacts. (b) xy projection map of the localized emission centers for the cluster with red kymograph trace, colorized by time frame. (c) xy projection map of the localized emission centers for the 100 x 100 nm^2 microgel area with cyan kymograph trace, colorized by time frame.

REFERENCES

1. Huang, F.; Sirinakis, G.; Allgeyer, E. S.; Schroeder, L. K.; Duim, W. C.; Kromann, E. B.; Phan, T.; Rivera-Molina, F. E.; Myers, J. R.; Irnov, I.; Lessard, M.; Zhang, Y.; Handel, M. A.; Jacobs-Wagner, C.; Lusk, C. P.; Rothman, J. E.; Toomre, D.; Booth, M. J.; Bewersdorf, J., Ultra-High Resolution 3D Imaging of Whole Cells. *Cell* **2016** *166* (4), 1028-1040.

2. Lagnoux, D.; Delort, E.; Douat-Casassus, C.; Esposito, A.; Reymond, J. L., Synthesis and esterolytic activity of catalytic peptide dendrimers. *Chemistry* **2004,** *10* (5), 1215-26.

3. Dag, A.; Jiang, Y.; Karim, K. J.; Hart-Smith, G.; Scarano, W.; Stenzel, M. H., Polymer-albumin conjugate for the facilitated delivery of macromolecular platinum drugs. *Macromol Rapid Commun* **2015,** *36* (10), 890-7.

4. Benoist, E.; Loussouarn, A.; Remaud, P.; Chatal, J.-F.; Gestin, J.-F., Convenient and Simplified Approaches to N-Monoprotected Triaminopropane Derivatives: Key Intermediates for Bifunctional Chelating Agent Synthesis. *Synthesis* **1998,** *1998* (08), 1113-1118.

5. Urankar, D.; Košmrlj, J., Preparation of diazenecarboxamide–carboplatin conjugates by click chemistry. *Inorganica Chimica Acta* **2010,** *363* (14), 3817-3822.

6. Kumar, Y. P.; Das, R. N.; Schutte, O. M.; Steinem, C.; Dash, J., Bis-triazolyl diguanosine derivatives as synthetic transmembrane ion channels. *Nat Protoc* **2016,** *11* (6), 1039-56.

7. Hirokawa, Y.; Okamoto, T.; Kimishima, K.; Jinnai, H.; Koizumi, S.; Aizawa, K.; Hashimoto, T., Sponge-like Heterogeneous Gels: Hierarchical Structures in Poly(N-

isopropylacrylamide) Chemical Gels As Observed by Combined Scattering and Confocal Microscopy Method. *Macromolecules* **2008,** *41* (21), 8210-8219.

8. Acciaro, R.; Gilányi, T.; Varga, I.; Preparation of Monodisperse Poly(N-isopropylacrylamide) Microgel Particles with Homogenous Cross-Link Density Distribution, *Langmuir* **2011**, 27 (12), 7917-7925.

9. Wu, X. ; Pelton, R. H. ; Hamielec, A. E. ; Woods, D. R.; McPhee, W.; The kinetics of poly(N-isopropylacrylamide) microgel latex formation. *Colloid Polym Sci* **1994**, 272, 467.

10. Huang, B.; Wang, W.Q.; Bates, M.; Zhuang, X.W. Three-dimensional super-resolution imaging by stochastic optical reconstruction microscopy. *Science* **2008**, 319, 810-813.

11. Zhang, Z.; Kenny, S.J.; Hauser, M.; Li, W.; and Xu, K. Ultrahigh-throughput single-molecule spectroscopy and spectrally resolved super-resolution microscopy. *Nature Methods* **2015**, 12, 935 – 938.
